# Effects of Conformity Tendencies on Farmers’ Willingness to Take Measures to Respond to Climate Change: Evidence from Sichuan Province, China

**DOI:** 10.3390/ijerph191811246

**Published:** 2022-09-07

**Authors:** Junqiao Ma, Wenfeng Zhou, Shili Guo, Xin Deng, Jiahao Song, Dingde Xu

**Affiliations:** 1 College of Management, Sichuan Agricultural University, Chengdu 611130, China; 2 School of Economics, Southwestern University of Finance and Economics, Chengdu 610074, China; 3 College of Economics, Sichuan Agricultural University, Chengdu 611130, China; 4 Sichuan Center for Rural Development Research, College of Management, Sichuan Agricultural University, Chengdu 611130, China

**Keywords:** climate change, adaptive behavior, peer effects, mechanism analysis, China

## Abstract

Encouraging farmers to respond to climate change is very important for agricultural production and environmental governance. Based on the data of 540 farmers in Sichuan Province, China, the effects of conformity tendencies on farmers’ adaptive behavior decisions to climate change were analyzed using the binary logistic model and propensity score matching method (PSM). The results show that (1) relatives’ and friends’ adaptive behaviors to climate change positively affect farmers’ adaptive behaviors to climate change. (2) Compared with relatives and friends who do not visit each other during the New Year (weak ties), the climate change adaptation behavior of relatives and friends who visit each other during the New Year (strong ties) has a more significant impact on the climate change adaptation behavior of farmers. (3) Farmers with higher education levels and agricultural products without disaster experience are more significantly affected by peer effects and more inclined to take measures to respond to climate change. (4) Social networks and social trust play a partially mediating role in the peer effects of farmers’ adaptation to climate change, but there are differences between relatives and friends with different strong and weak ties.

## 1. Introduction

Climate change is related to the sustainable development of human beings and has become one of the most severe challenges facing human beings today [1,2]. According to the Sixth Assessment Report of the Intergovernmental Panel on Climate Change (IPCC), the global temperature may exceed 1.5 °C in the next 20 years. There is no doubt that the global climate shows a gradual warming trend [3]. Correspondingly, extreme weather events increase, agricultural and meteorological disasters such as droughts and floods occur more frequently [4], ecological deterioration, sea level rise, water resource stress, and other problems emerge endlessly [5]. They will all affect the ecosystem, economic system, and social system of all countries [6]. Among them, agricultural production is more vulnerable to climate change [7], which is more evident in developing countries. According to statistics, the direct economic loss caused by meteorological disasters in China accounts for 3~6% of GDP, and the affected farmland area reaches more than 50 million hm^2^ [8]. In the next 10 years, climate change will lead to an average reduction of 5~10% in the total output of major crops in China [9].

Rural areas are essential for agricultural production in developing countries [10]. However, the capacity of rural agricultural production in developing countries to respond to climate change is still limited [11]. As an important part of agricultural production, whether farmers take measures to respond to climate change will affect the choice of agricultural production mode and the stability of the agricultural system [12]. However, some studies show that farmers’ perceptions and responses to climate change are not optimistic at present [13]. For example, studies have found regional differences in farmers’ perception of climate change, and their perception effectiveness needs to be improved [14]. Meanwhile, in some areas of China, more than half of farmers have not actively responded to climate change [15]. Therefore, how to solve the problems of farmers’ low enthusiasm and inadequate ability to adapt to climate change has become the only way to promote the transformation of modern agriculture and global environmental governance.

Adaptation to climate change refers to the process in which people try to avoid the damage to personal life and property caused by climate change while taking advantage of the positive effects of climate change [16]. The academic community has conducted extensive studies on adaptation to climate change, mainly involving willingness to cope with climate change, adaptive behavior of climate change, and climate change perception [17,18]. Among them, the adaptive behavior of climate change and its driving mechanism has been the focus of academic and political attention. From the existing research, the academic research on the influencing factors of adaptive behavior of climate change mainly focuses on the following aspects. First, basic family characteristics, such as farming year, gender, and age [19,20] of farmers, have been confirmed to be positively and significantly correlated with farmers’ adaptive behavioral decisions. The second is the perception level. Some studies find that risk perception [21] and ecological perception [22] will promote farmers to respond actively to climate change, while economic perception will hinder farmers from taking response measures [23]. Third, family capital endowment, including human capital, financial capital, social capital, natural, geographical environment endowment, and so on; for example, Below et al. [24] and Mugi-Ngenga et al. [25] found that planting income, household annual net income and household land area in economic capital were positively and significantly correlated with farmers’ adaptation to climate change. In addition, natural and environmental endowments such as market distance and agroecological zone also affect farmers’ choice of adaptation mode to climate change.

To sum up, although a lot of existing research focuses on the key factors influencing farmers’ climate change adaptation behavior, these factors are mainly focused on family and personal characteristics, cognitive level, and family capital endowment without visual angles from the same group of effect research, focus on the strength of social relation network relations for climate change adaptation behavior of farmers (peer effects refers to the impact of peer group behavior on individual behavior choices [26]). In vast rural areas, especially in developing countries, farmers have limited access to information due to traffic, terrain, and other factors, so they often refer to other individuals’ decisions [27]. Neighbors are important reference objects for farmers [28]. At present, some scholars have applied peer effects analysis methods to research fields such as agricultural technology adoption, clean energy use, agricultural insurance purchase, and so on. For example, Liu et al. [29] found that farmers increased their understanding of technology through observational learning and communication with neighbors. Wang et al. [30] showed that the expansion of informal social networks might harm farmers’ conformity, while the improvement of formal social networks and interpersonal trust will bring positive benefits, both of which will further influence farmers’ adaptive behavior decisions on climate change. However, in the context of global warming, the peer effects and their driving effect on farmers’ adaptation to climate change is still a relatively neglected issue. So, are peer effects affecting farmers’ adaptive behavior decisions on climate change? If so, do relatives and friends with different strong and weak ties have different impacts on farmers’ adaptive behaviors to climate change? This is the question to be solved in this study.

## 2. Materials and Methods

### 2.1. Data Sources

Sichuan Province is one of the main grain-producing areas in China, with complex and diverse terrain. The western area of Sichuan Province is a mountainous plateau, more than 4000 m above sea level. The eastern part is a hilly plain, with an elevation between 1000 and 3000 m. Due to topographic reasons, the natural conditions of Sichuan Province are quite different, forming a variety of climates, such as the alpine climate and subtropical monsoon climate. In addition, the level of regional economic development in Sichuan Province is also different. In general, the economic development of the Chengdu plain area is good, but the economic development of the mountainous area is poor. As a typical climate-vulnerable area, Sichuan Province is prone to agricultural meteorological disasters such as drought, flood, and frost. Therefore, the data used in this study are from the statistical summary of the field survey conducted by the research group in July 2021 in Sichuan Province, China. The survey method is the one-to-one interview. The respondents were family members who were familiar with family agricultural production and assets, and 61.30% of them were household heads. The research content mainly includes the basic characteristics of farmers, climate change perception, climate change response situation, etc. Some objective indicators, such as annual household income, mainly take 2020 as the anchor point, and some subjective indicators, such as risk perception, mainly investigate the current cognition of respondents [31]. In order to ensure the representativeness of samples, stratified equal probability sampling is adopted to determine the research samples. The specific process is as follows.

**Figure 1:**
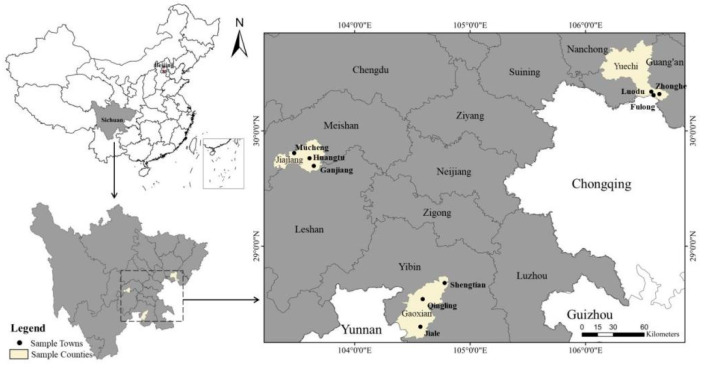
Location map of sample counties and towns.

**Figure 2:**
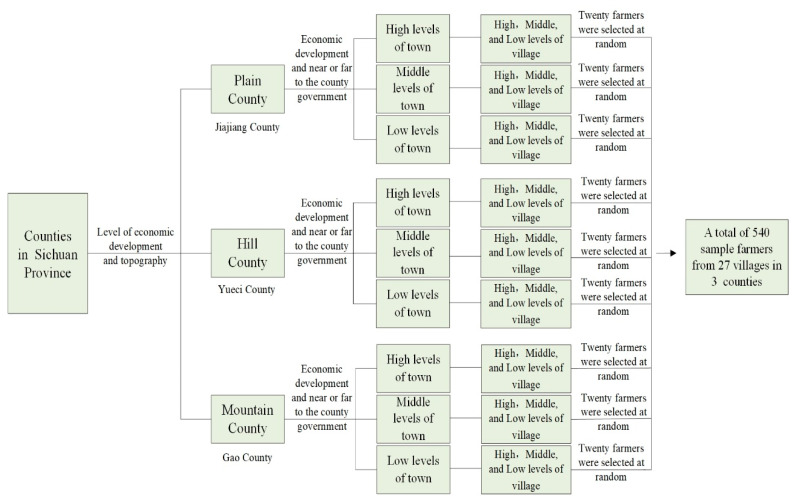
Survey sampling process.

First, the 183 counties in Sichuan Province were divided into plain, hilly, and mountain areas according to the local landforms (plain, mountain, and hill) index, and one district and county were selected from each. Second, using the indexes to determine the distance between the towns and the county government and the level of economic development, three sample towns of good, medium, and poor in each district and county were randomly selected, and a total of nine sample towns were selected. Third, according to the distance of villages to the town government and the level of economic development, each sample village was randomly selected as good, medium, and poor villages, totaling 27 sample villages. Then, the members of the research group contacted the village cadres in advance and selected 20 farmers from the village roster according to the preset random number table as the investigation object. Finally, 540 effective peasant household questionnaires were obtained from 9 townships and 27 villages in 3 districts and counties (Figure 1 and Figure 2).

### 2.2. Theoretical Analysis and Research Assumptions

The term peer effects refers to the fact that people live in a peer group composed of individuals, and the choices of individuals are affected by the choices of other individuals in the circle [32,33]. In daily life, in order to reduce the decision-making risk caused by incomplete information, people tend to learn from other people’s decisions or behaviors to reduce uncertainty [34,35]. At present, relevant theories of peer effects are applied to the investment decisions of enterprises [36], clean energy use [37], and social education [38], and they have been widely used in many fields. At the same time, Chinese scholars combined traditional customs and further localized the “peer group circle”. Sociologist Fei Xiaotong believes that individual social relations exist in multiple circles, which are relatively independent and partially integrated [39]. Due to the influence of the traditional concept of “family culture”, the individual social network circle usually develops on the basis of blood relationships and geographical relationships. On this basis, Yang [40] divided Chinese people’s networks into family relationships, acquaintance relationships, and stranger relationships according to different degrees of closeness and estrangement. The relationship based on blood relationship and geography is more closely related to individuals and has a more significant impact on individual decision-making [41]. Many studies have shown that incomplete information is an important cause of peer effects [42]. Therefore, in rural areas with backward economies and occluded topography, farmers tend to converge with others under the condition of limited information channels. Among them, consanguinity and geo-related groups are the main objects of convergence [40]. Although with the acceleration of urbanization, the original relationship network in China’s rural areas shows a changing trend [43], on the whole, the behavioral choices of farmers are still closely related to the decisions of relatives and friends [27]. Therefore, this paper takes family and friends as reference objects to study the peer effects of farmers’ adaptive behaviors to climate change.

In fact, there are differences in the relationships between friends and relatives. In 1973, Granovetter proposed the “strong and weak relationship theory” and elaborated related classification concepts on the basis of social network theory. Strong ties refer to a social relationship with a large amount of emotional investment and frequent interaction and communication. Weak ties usually exist between friends and relatives with low reciprocal exchange frequency and limited intimacy. In the strong and weak relationship hypothesis, the strong ties have higher information homogeneity, while the weak relationship has richer information channels and levels [44]. Since then, the strong and weak relationship theory has been mostly applied in enterprise operation [45], knowledge sharing [46], and population mobility [47]. However, the strong and weak relationship theory seems to have more general applicability. Zhang and Zhou [48] compared the effects of kinship and geographical networks on the adjustment of farmers’ planting structure and found that the will of farmers with whom they had strong ties had a greater impact than those with weak ties. Therefore, there may also be a circle effect in the adaptation behavior of farmers to climate change. However, in the existing literature, the influence differences of peer effect under strong and weak ties are often ignored. Compared with strong ties groups among family and friends, weak relationship groups have lower information circulation rates [49], which has a less direct impact on farmers’ information acquisition and climate change adaptation behavior.

In real life, social trust is an important reason for the peer group effect [50]. In rural areas with limited communication channels, other farmers often become important objects for farmers to obtain information. The trust relationship formed in the long-term communication between farmers will affect the individual behavior choice of farmers. On the one hand, when some farmers passively respond to climate change or blindly adapt to climate change by applying a lot of chemical fertilizers, it may lead to blind obedience of farmers in responding to climate change. On the other hand, the higher the trust in farmers to relatives, friends, and strangers, the higher the information acceptance of farmers. When individuals make behavioral choices in response to climate change, they can have a more comprehensive understanding of the relevant situation, making it easier to choose scientific and effective ways to adapt to climate change [51]. In addition, in the process of long-term trust, interaction, and communication between farmers, a relatively stable social network will gradually be formed [52]. When farmers have a more comprehensive social network and are more closely connected with other farmers, the frequency and quality of information dissemination may be higher. Finally, farmers have a deeper understanding of climate change risks and responses and are more inclined to adapt actively to climate change [53].

Based on this, the following hypotheses are made in this study.

**Hypothesis** **1** **(H1).** *The adaptive behaviors of family and friends to climate change will positively affect the adaptive behaviors of farmers to climate change*.

**Hypothesis** **2** **(H2).** *Compared with weak relatives and friends, the adaptive behaviors of strong relatives and friends have a greater impact on farmers’ adaptive behavior decisions to climate change*.

**Hypothesis** **3** **(H3).** *Social trust plays a mediating role in the co-group effect (convergence among relatives and friends, strong relatives and friends, and weak relatives and friends) of farmers’ adaptation to climate change*.

**Hypothesis** **4** **(H4).** *Social networks play a mediating role in the co-group effect (convergence among relatives and friends, strong relatives and friends, and weak relatives and friends) of farmers’ adaptation to climate change*.

### 2.3. Variable Definitions

The purpose of this paper is to explore the peer effects on farmers’ decision-making on climate change. According to the concept of “adaptive behavior with purpose” proposed by Smit et al. [54], combined with the research of Lv and Chen [55] and Tong et al. [56], farmers’ adaptation measures to climate change are divided into active adaptation and passive adaptation. The former refers to the characteristics of farmers’ adaptive behavior to climate change with active adaptation beforehand, including adjusting seed types/varieties, building infrastructure, learning climate change-related technologies, and going out to work because of climate change. The latter refers to the passive ex-post adaptation of farmers’ climate change adaptation behaviors, including increasing pesticide/fertilizer use, adjusting irrigation, and adjusting farming time. In the question “Are you taking action because of climate change?”, if the farmer does not adopt either of the two measures, it is marked as “no” and assigned a value of 0. If either or both measures are used, it is marked “yes” and assigned a value of 1. Among the data from 540 farmers, 489 households, accounting for 90.74% of the total, have adopted measures to cope with climate change. According to Ma [57] et al., it is more in line with the current situation of Chinese society to evaluate the social network of residents through whether they visit each other during the New Year. Therefore, in this study, “relatives and friends who visit during the New Year” are defined as strong ties, and “relatives and friends who do not visit during the New Year” are defined as weak ties. The core independent variables of this study are “Whether relatives and friends take measures to deal with climate change”, “Whether relatives and friends who visit during the New Year take measures to deal with climate change”, and “Whether relatives and friends who do not visit during New Year take measures to deal with climate change”. Table 1 shows the results of the correlation analysis of core variables. The results showed that the three core independent variables were positively correlated with the dependent variable.

In addition, considering that farmers’ decision-making on coping with climate change may be affected by a variety of other factors, this paper included the individual characteristics of respondents, family characteristics, climate perception, and meteorological disaster experience into the model as control variables. According to the studies of Feng et al. [58], individual characteristics of the respondents include gender, age, education level, and other indicators of the respondents, which are often considered to be significantly correlated with farmers’ climate change response behaviors. According to the research of Lv and Chen [55], household characteristics mainly include human capital indicators such as the number of the household labor force and per capita gross income. An economic capital index includes household per capita cultivated land area and geographical and environmental endowment indicators such as distance from home to market and fertility of cultivated land. Wheelers et al. [59] believed that farmers’ perception of climate risk had an important impact on their decision-making about climate change adaptive behavior. Therefore, the question, “How worried are you about climate change” is included in the control variable in this paper. Zhang et al. [60] believe that farmers’ risk perception can be divided into four categories: perception of individuality, perception of production, perception of cost, and perception of severity. Therefore, this paper focuses on these four dimensions. “How serious do you think climate change poses a threat to individuals?”, “Are you worried about the serious impact of climate change on agricultural production?”, “Are you worried about the serious impact of climate change on the safety of life and property?”, and “Are you worried about the serious impact of climate change on your life?” were incorporated into the control variables for assessment. Song and Shi [61] and Sun [62] found that the frequency and trend of extreme meteorological events and the duration of local residence also affected the decision-making of farmers’ sexual behavior in response to climate change. Therefore, in this paper, “Have crops been damaged by the weather” and the time interviewees live in the village are also included in the model as control variables. Finally, in order to reduce the influence of regional differences on the regression analysis results, corresponding regional dummy variables are added. The definition and basic statistics of variables are shown in Table 2.

### 2.4. Research Methods and Models

#### 2.4.1. Binary Logistic Regression

Considering that the dependent variable in this paper, namely whether farmers take measures to cope with climate change, is a dichotomous variable, this study attempts to construct a binary logistic econometric model to explore the peer effects of family and friends on farmers’ adaptive behavior to climate change. The model formula is as follows:

(1) $$\mathrm{logit}\left(p\right)=\ln(\frac{p}{1-p})=\beta_{0}+\beta_{1}X_{1}+\cdots +\beta_{i}X_{i}+\varepsilon$$

In Formula (1), p is the probability that farmers are willing to take countermeasures against climate change; $\beta_{0}$ is a constant term; $X_{1}\cdots X_{i}$ is the independent variable, including the core independent variable, control variable, and regional dummy variable; $\beta_{1}\cdots \beta_{i}$ is the regression coefficient; ε is the residual term. Among them, there are three core independent variables, including whether relatives and friends have taken measures to deal with climate change, whether relatives and friends who have visited each other during the New Year have taken measures to deal with climate change, and whether relatives and friends who have not visited each other during the New Year have taken measures to deal with climate change.

#### 2.4.2. Propensity Score Matching Method (PSM Model)

This paper focuses on the differences in the effects of family and friends on farmers’ adaptive behavior decisions of climate change under different strong and weak ties, which can be achieved by comparing the adaptive behavior decisions of farmers under the two conditions of family and friends taking measures against climate change and not taking measures against climate change. However, farmers’ responses to climate change may be self-selected, and different family resource endowments may also affect farmers’ behaviors, leading to selection bias. At the same time, since it is impossible to observe the impact of relatives and friends who take measures to cope with climate change on farmers when they do not take measures to cope with climate change, they can only observe the current coping behavior of families to cope with climate change, and the absence of observation data will lead to deviation and biased estimation of samples. Therefore, this paper introduces the propensity score matching (PSM) proposed by Rosenbaum and Rubin (1983) to solve the problem and improve the robustness of the results. The general idea of PSM error correction is as follows. Whether the sample, according to the relatives and friends of climate adaptation measures, is divided into an experimental group and control group, and then according to a certain way match, on the outside of the control conditions of the same cases by judging the experimental group and control group on climate change adaptation farmers to analyze the difference between friends and relatives to cope with climate change behavior of farmers the impact of climate change managing behavior decision-making. The specific operation process is as follows. First, the propensity score is calculated by the logistic regression model. Secondly, according to the score, the experimental group and control group were matched with an appropriate algorithm. Finally, the average willingness (ATT) of the experimental group and the control group to adapt to climate change was calculated as follows:

(2) $$\mathrm{ATT}=E\left[(y_{1i}-y_{0i}\right){|D}_{i}=1]=E\left(y_{1i}{|D}_{i}=1\right)-E(y_{0i}|D_{i}=1)$$

In Formula (2), $D_{i}$ is a binary variable, i represents whether farmers belong to the experimental group. $D_{i}=1$ indicates that farmers belong to the experimental group; otherwise, it is the control group. $y_{1i}$ and $y_{0i}$ represent the estimation results of the experimental group and the control group, respectively. ATT  said friends and family to climate change adaptation measures the family I response to the situation of $E(y_{1i}|D_{i}=1)$ with family and friends did not respond to climate change response to the situation of $E(y_{0i}|D_{i}=1)$. Due to $E(y_{0i}|D_{i}=1)$ can not be observed, PSM through algorithm will $E(y_{0i}|D_{i}=1)$ substitute for $E(y_{0i}|D_{i}=0)$.

#### 2.4.3. Mediation Effect Model

In this paper, the mediating effects are used to explore the process of quantifying the peer effects on farmers’ adaptation to climate change. The mediating effect refers to the direct influence of X on Y and the indirect influence of X on Y through variable M during the impact of independent variable X on dependent variable Y. Its model is expressed as follows:

(3) $$Y=\mathrm{cX}+\varepsilon_{1}$$

(4) $$M=\mathrm{aX}+\varepsilon_{2}$$

(5) $$Y=\;\beta \prime \;X+\gamma M+\varepsilon_{3}$$

In Formulas (3)–(5), Y represents the climate change adaptation behavior of farmers, M represents the social network and social trust, X represents the climate change adaptation behavior of relatives and friends. c a γ  are the estimation parameters, respectively, and $\varepsilon_{1}{\;\varepsilon }_{2}{\;\varepsilon }_{3}\;$ are the residuals. Formula (3) represents the total effect of family and friends’ adaptive climate change behaviors on farmers’ adaptive climate change behaviors. Formula (4) represents the process of the impact of family and friends’ coping with climate change on social networks and social trust. Formula (5) represents the process of social networks and social trust in farmers’ adaptation to climate change.

## 3. Results and Discussion

### 3.1. Binary Logistic Model Estimation

Table 3 shows the regression results of family and friends’ coping behaviors to climate change on farmers’ adaptive behavior decisions to climate change. The reported results are the marginal effect of the binary logistic model and the standard error of cluster at the county level. Models 1, 3, and 5 only included the core variables, the coping behaviors of relatives and friends, relatives and friends with and without New Year’s visits to climate change. In models 2, 4, and 6, the influence of control variables and regional dummy variables was considered on the basis of including corresponding core variables. As shown in the table, whether control variables and regional dummy variables are added or not, relatives and friends, relatives and friends who visit each other during the New Year (strong ties), and relatives and friends who do not visit each other during the New Year (weak ties), all have a significant impact on farmers’ adaptation behavior decisions to climate change.

Compared with models 1, 3, and 5, models 2, 4, and 6 have more comprehensive consideration and better effects. Therefore, the regression results of models 2, 4, and 6 are mainly analyzed. In order to better explain the regression results, we tested the marginal effect of each variable in the estimated results of models 2, 4, and 6. First of all, the regression results of model 2 show that family and friends have a significant positive impact on farmers’ behavioral decisions on adaptation to climate change. Every 1% increase in family and friends’ response measures to climate change, the probability of farmers’ response measures to climate change will increase by 21.1%. This phenomenon may be because rural China is an acquaintance society, and farmers’ coping behaviors in ecological environment construction are more easily influenced by others [63]. Secondly, in the regression results of model 4, we found that relatives and friends who visit during New Year also have a significant positive impact on farmers’ climate change adaptation behavior. For every 1% increase in the number of family and friends who visit each other, the probability of farmers taking measures to cope with climate change will increase by 20.0%. Compared with the conformity of family and friends, the influence of family’s behavior choice is more obvious. This is in line with our hypothesis that compared with relatives and friends, relatives and friends who have New Year visits are more closely connected with farmers, which has a greater impact on farmers’ behavior and decision-making. Finally, the regression results of model 6 show that the behaviors of family and friends who do not visit during the New Year also have a significant positive impact on the behavioral decisions of farmers’ adaptation to climate change and the probability of farmers’ adaptation to climate change will increase by 15.1% when the relatives and friends take measures to climate change increase by 1%. This may be because farmers rely more on and communicate more frequently with relatives and friends who do not have New Year’s visits, although they can provide diversified information and expand the scope of dissemination. Therefore, the influence of no New Year visit is less than that of friends and relatives who visit each other during the New Year [49]. In addition, in terms of control variables, gender, age, and length of residence have a significant negative impact on farmers’ adaptation to climate change, and annual cash income per capita and per capita arable land area has a significant positive impact on farmers’ adaptation to climate change.

The above results indicate that relatives and friends, relatives and friends with and without New Year visits, all have a significant positive impact on farmers’ climate change response measures. However, this may be the result of self-selection by the surveyed farmers. Therefore, PSM proposed by Rosenbaum and Rubin (1983) is adopted in this paper to avoid selection bias.

### 3.2. Estimation Results of PSM

First of all, the logistic model was constructed to predict the probability of conformity of farmers’ climate change response behavior to their relatives and friends, relatives and friends with and without New Year’s visit. Then PSM was used to match the propensity score. Since the academic circle has not reached a consensus on the most matching algorithm, this paper chooses 1:4 nearest neighbor matching, radius matching, and kernel matching to comprehensively evaluate the results.

**Figure 3:**
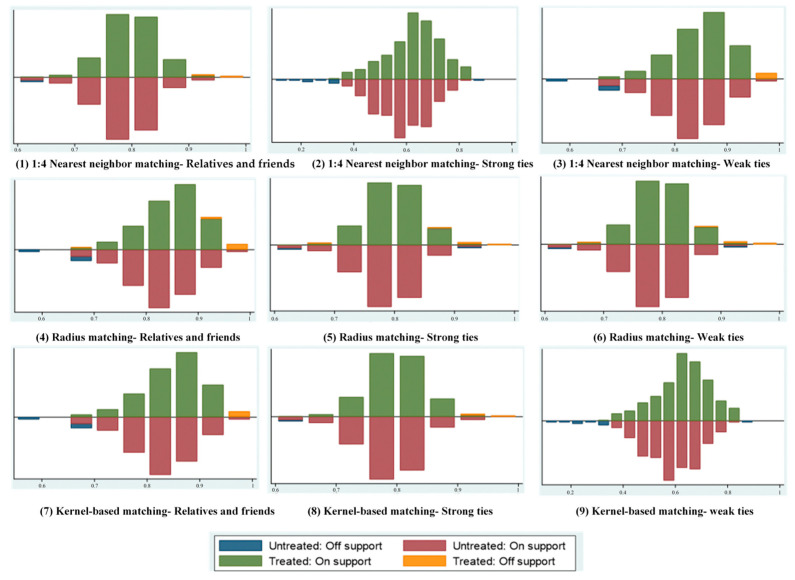
Influence of cohort effect after bias correction.

Secondly, the matching effect is evaluated by the standard support test. As shown in Figure 3, the propensity score distribution of farmers with convergence tendency and farmers without convergence tendency is different in the adaptation behavior of farmers to climate change. After appropriate matching by the corresponding algorithm, the difference between the experimental group and the control group is weakened, and the sample distribution effect is better.

Finally, the average treatment effect is calculated. This paper explores the impact of family and friends’ response to climate change on farmers’ response to climate change, focusing on the change in farmers’ response to climate change whose family and friends take response measures to climate change. Therefore, the mean treatment effect (ATT) of the experimental group was used for analysis. Table 4 presents the estimation results of the average processing effects of 1:4 nearest neighbor matching, radius matching, and kernel matching. SE in the table is the standard error obtained by the self-help method repeated 500 times. ATT’s results show that, even after the observed systemic differences between samples are avoided, the climate change adaptation of family and friends still has a significant impact on farmers’ climate change adaptation behavior. This confirms the previous binary logistic model regression results.

Specifically, for friends and family, ATT was positively significant at 5% for all matching results. Among them, the ATT value obtained by radius matching is the largest, which is 0.446. The second is 1:4 nearest neighbor matching, which is 0.433. Kernel matching, 0.376. The results show that the convergence of relatives and friends among farmers increases the likelihood of responding to climate change. In terms of friends and relatives who will visit each other during the New Year, all the matching results estimated ATT was not significant. The results show that the convergence of farmers’ friends and relatives with New Year’s visits has no obvious effect on their willingness to adapt to climate change. For friends and relatives without New Year’s visits, all the matching results estimated significant positive ATT at a 1% level. Among them, the ATT value obtained by nuclear matching is the largest, which is 0.183. Additionally, 1:4 nearest neighbor matching and radius matching results are similar, which are 0.175 and 0.173, respectively. The results show that the convergence of farmers with friends and relatives without New Year visits increases their willingness to adapt to climate change.

### 3.3. Heterogeneity Analysis

It has been verified above that the climate change adaptation behavior of relatives and friends (including relatives and friends who visit during the New Year and those who do not visit each other during the New Year) will affect farmers’ climate change response behavior. Next, this paper will discuss whether the climate change adaptive behaviors of relatives and friends (including those with and without New Year’s visits) will have a different impact on the climate change adaptive behaviors of farmers. According to the research of Lv and Chen [55], a certain level of education will improve farmers’ innovation ability and observation ability, and farmers with higher education are more willing to take the initiative to adapt to climate change. The experience of disaster will make farmers’ perception of climate change more noticeable, thus having an indirect positive impact on farmers’ behavioral decisions on adaptation to climate change [64]. Therefore, this paper divides families into groups from two dimensions, the education level and crop disaster experience. Among them, the education level of the respondents is divided into two groups according to whether the respondents have an education level below primary school or above primary school (Table 5).

1. The education level of the respondents. The results show that the impact of relatives and friends (including those who visit during New Year and those who do not visit during New Year) on climate change adaptation behavior of farmers with higher education levels is more significant. Respondents who have a primary school education or above are more influenced by relatives and friends. The possible explanation is that farmers with higher education have more information channels and pay attention to diversified learning knowledge. The influence of New Year visits to relatives and friends is more obvious in respondents who only have a primary school education. This may be because farmers with lower levels of education have less access to information and limited judgment and may rely more on learning from close relatives and friends.

2. Crop disaster experience. From the results, it can be seen that the families with relatives and friends (including relatives and friends who visit during the New Year and those who do not visit during the New Year) who have no experience of agricultural products disaster have a greater impact on climate change response measures. The possible reason is that the farmers who have not experienced a disaster are not experienced enough and tend to refer to the opinions of relatives and friends. However, families with agricultural products affected by disasters have higher cognitive abilities and are less affected by relatives and friends when facing the risk of climate change.

### 3.4. Mechanism Analysis

The above results indicate that farmers’ adaptive behavior decisions on climate change have a circle effect. Next, this paper analyzes the mediation effect from two mechanisms: peer effects → social network → response to climate change, peer effects → social trust → response to climate change. According to the research of Wang and Guo [65], the social network relationship of farmers can be expressed as the communication and interaction between farmers and other subjects, among which the following ceremony is an important way of social communication in rural areas. Therefore, this paper uses the indicators of “household gift expenditure in 2020” and “gift money received by relatives and friends in 2020” to reflect farmers’ social networks. Secondly, farmers’ trust also has a specific influence on their behavior choices. Zhang [66] finds that trust in the majority of people and trust in the government is a vital link. Therefore, based on the reference of existing studies, this paper analyzes the trust of farmers from the two dimensions of “trust in local government” and “trust in strangers” (Table 6).

The regression results of mechanism 1 show that the effects of the two paths are not good: relatives and friends → gift money received by relatives and friends in 2020 → response to climate change and visiting relatives and friends on New Year’s Day → gift money received by relatives and friends in 2020 → response to climate change. In model 1, climate change response behaviors of relatives and friends, relatives and friends with or without New Year visits significantly affect farmers’ social networks at the levels of 10%, 1%, and 5%. In model 2, climate change coping behavior without visiting relatives and friends has a negative and significant impact on farmers’ social networks at a 5% level. This phenomenon may be caused by the fact that farmers tend to take measures to cope with climate change out of the psychology of avoiding risks and reducing losses when they know the experience of their relatives and friends (including relatives and friends who visit during the New Year and those who do not) about coping with climate change. For the relatives and friends who do not pay New Year’s visits, farmers have less human contact with them. Therefore, if the influence of the peer effect is measured only by the gift money from friends and relatives in the social network, the influence of family and friends who do not pay New Year’s visits will be small and even negative.

The regression results of mechanism 2 show that only relatives and friends → trust of government → response to climate change, relatives and friends → trust of strangers → response to climate change and visiting relatives and friends on the New Year → trust of strangers → response to climate change have good effects. In model 3, the behaviors of family and friends to cope with climate change at a 1% level significantly affect farmers’ trust in the government. In model 4, relatives and friends and those with New Year visits have a significant negative impact on farmers’ trust in strangers at the level of 5% and 10%, respectively. When the mediating variable of “social trust” is added, the influence coefficients of family and friends and relatives with New Year visits on farmers’ adaptation to climate change increase from 3.687 and 3.481 to 3.824 and 3.746. The reason for this phenomenon may be that, for relatives and friends, government publicity and relevant policies may increase their awareness of climate change and thus enhance their willingness to take measures against it. Although they interact less frequently with strangers, they still acquire a broader range of knowledge. Finally, the influence of family and friends on farmers’ adaptation to climate change will be enhanced.

### 3.5. Robustness Test

In this paper, a robustness test is carried out on the basis of the benchmark model, and the results are shown in Table 7. The Logit model was used to regress the sample data into three groups: Gaoxian, Jiajiang, and Yuechi county. According to the comparison of regression results, in Jiajiang and Yuechi county, the impact of climate change adaptation behavior of relatives and friends (including strong ties and weak ties) on farmers’ response to climate change is positive and significant at a 1% level. In Gaoxian county, the impact of climate change adaptation behavior of relatives and friends with weak ties on farmers’ response to climate change was positive and significant at a 5% level, while the impact of relatives and friends with strong ties was significant at a 1% level. In general, the results of the model are basically consistent with the results of the model in Table 3 in terms of trend, and most of them are differences in coefficients. This indicates that regional differences have no obvious influence on the study, and the same peer effects have a universal effect on the adaptation behavior of farmers to climate change, and the results are basically robust.

## 4. Conclusions

Based on the analysis of the possible circle effect from the perspective of strong and weak ties, the influence and mechanism of the peer effects on farmers’ adaptive behavior decision of climate change were analyzed using the binary logistic model and PSM method (propensity score matching method). The results show that (1) relatives’ and friends’ adaptive behaviors to climate change positively affect farmers’ adaptive behaviors to climate change. (2) Compared with relatives and friends who do not visit each other during the New Year (weak ties), the climate change adaptation behavior of relatives and friends who visit the New Year (strong ties) has a greater impact on the climate change adaptation behavior of farmers. (3) Farmers with higher education levels and agricultural products without disaster experience are more significantly affected by the peer effects and more inclined to take measures to respond to climate change. (4) Social networks and social trust play a partially mediating role in the peer effects of farmers’ adaptation to climate change, but there are differences between relatives and friends with different strong and weak ties.

Compared with existing studies, the marginal contributions of this paper are as follows. First, considering the impact of the peer effects on the adaptive behavior of farmers to climate change, the possible circle effect is analyzed from the perspective of strong and weak ties (there are differences between strong and weak relationship relatives and friends in the response of farmers to climate change. The driving effect of strong ties relatives and friends on the adaptation of farmers to climate change is greater than that of weak ties relatives and friends). This study verified that the theory of the peer effects and strong/weak ties might also be applicable to farmers’ behavior choices to adapt to climate change in rural areas of China. Second, the PSM method (propensity score matching method) was used to improve the accuracy of matching results and correct the endogeneity problem caused by self-selection bias in farmers’ climate change response behavior. Third, through heterogeneity analysis, the paper tested whether the effect of the same group effect was constrained by the conditions of farmers’ education level and agricultural disaster experience and the degree of restriction. Fourthly, the mediating effect model was used to deeply analyze the mechanism of “Social trust” and “Social network” in the decision-making of farmers’ climate change adaptation behavior.

There are still some deficiencies in this study, which can be further improved in future research. For example, the types of climate change response measures of farmers can be further divided according to the disaster characteristics of different regions (such as drought and flood). Secondly, family cultivated land area, family income level, and other factors may also influence the driving process of family and friends’ response to climate change and farmers’ response to climate change. Finally, the mediators such as social trust and social networks can be further explored in various ways (such as social reciprocity and social learning) in the peer group effect of farmers’ climate change adaptive behaviors.

## 5. Policy Recommendations

Climate change has become one of the major crises facing humankind. According to the IPCC assessment, climate change will intensify further in the coming decades, with extreme heat reaching critical tolerance thresholds for agricultural production and human health more frequently. Many studies have confirmed that climate change has a significantly negative impact on grain yield [7]. In order to face the challenge of climate change, China has proposed the “dual carbon goal” from the perspective of sustainable development (i.e., peak carbon dioxide emissions by 2030 and carbon neutral by 2060), demonstrating its status as a major responsible country in the construction of the global ecological environment. On the one hand, the Chinese government attaches great importance to climate change, and it has issued several provisions and directives to guide participants at all levels to respond to climate change actively. On the other hand, due to the limitation of education level and economic income, micro-individuals lack the understanding and coping ability to deal with climate change, and these problems are more obvious in rural areas. Therefore, how to improve farmers’ enthusiasm and ability to cope with climate change scientifically is related to national food security and the realization of the “dual carbon target”, as well as global ecological and environmental governance.

This study has important implications for guiding farmers to take measures against climate change, and its conclusions can also be applied to policy making in other developing countries. To be specific, (1) considering that the current understanding of climate change is still relatively limited, the state should encourage the academic community to strengthen the research on climate change. To fully understand the current situation of climate change and its impact on agricultural production, formulate policies and guidelines to address climate change and supervise the implementation of local governments at all levels. (2) Considering the positive impact of cohorts on farmers’ adaptation to climate change, local governments should implement the instructions of higher governments according to local conditions. At the same time, we should increase the training of farmers on climate change, expand the information channels and publicity channels in rural areas, and reasonably use the herd effect of relatives and friends to guide farmers. (3) Considering that communication and learning with friends and relatives can reduce decision-making uncertainty, the information brought by friends and relatives with weak ties is more comprehensive and has more reference value. Farmers should strengthen contact with relatives and friends, take an active part in local climate change publicity and exchanges, and improve their ability to cope with climate change through television, mobile phones, and other media.

## Figures and Tables

**Table 1:** Correlation analysis table of core variables.

Variables	Climate	Relatives and Friends	Strong Ties	Weak Ties
Climate	1.0000			
Relatives and friends	0.4484 ***	1.0000		
Strong ties	0.4412 ***	0.7406 ***	1.0000	
Weak ties	0.2418 ***	0.4480 ***	0.3583 ***	1.0000

Note: *** *p* < 0.01.

**Table 2:** Variable definitions and descriptive statistics.

Variable	Variable Measure	Mean	Standard Deviation
Climate	Are you taking action because of climate change? (0 = no, 1 = yes) ^c^	0.9074	0.29
Relatives and friends	Whether relatives and friends take measures to deal with climate change? (0 = no, 1 = yes) ^c^	0.8463	0.36
Strong ties	Whether relatives and friends who visit during New Year take measures to deal with climate change? (0 = no, 1 = yes) ^c^	0.7963	0.40
Weak ties	Whether relatives and friends who do not visit during New Year take measures to deal with climate change? (0 = no, 1 = yes) ^c^	0.6093	0.49
Gender	Gender of the respondents (0 = male, 1 = female)	0.1111	0.31
Age	Age of the respondents (year)	58.93	11.02
Education	Years of education of the respondents (year)	6.75	3.17
Labor	The proportion of the labor force aged 16–64 to total household population (%)	0.26	0.23
Income	Household per capita annual cash income in 2020 (RMB/person) ^a^	19,462.51	33,420.40
Land	Per capita arable land area in 2020 (land/person)	1.43	4.26
Distance	Distance from home to market (km)	3.31	2.60
Risk perception	How worried are you about climate change? (1–5) ^b^	3.85	1.17
Individuality perception	How seriously do you think climate change threatens you personally? (1–5) ^b^	3.52	1.21
Production perception	Are you worried about the serious impact of climate change on agricultural production? (1–5) ^b^	3.53	1.21
Cost perception	Are you worried about the serious impact of climate change on the safety of life and property? (1–5) ^b^	4.20	1.03
Severity perception	Are you worried about the serious impact of climate change on your life? (1–5) ^b^	3.80	1.14
Residence time	How long have you lived in this village? (year)	50.32	17.31
Disaster experience	Have crops been damaged by the weather? (0 = no, 1 = yes) ^c^	0.7019	0.46
County	Dummy variable of county (Yuechi = 0)		

Note: ^a^ During the survey period, USD 1 = RMB 6.74; ^b^ 1–5 are indicators measured using the 5-point Likert scale, which means from strongly disagree to strongly agree; ^c^ Among the dichotomous variables, the value “Mean” means that XX% respondents choose “yes”. For example, in the variable “Disaster experience”, 70.19% of respondents chose “yes”.

**Table 3:** Regression results of the binary logistic model.

	Model 1	Model 2	Model 3	Model 4	Model 5	Model 6
Relatives and friends	0.191 *** (0.027)	0.211 *** (0.008)				
Strong ties			0.197 *** (0.004)	0.200 *** (0.012)		
Weak ties					0.139 *** (0.031)	0.151 *** (0.016)
Gender		−0.029 ** (0.013)		−0.039 *** (0.011)		−0.043 *** (0.009)
Age		−0.002 ** (0.001)		−0.001 *** (0.000)		−0.003 ** (0.001)
Education		−0.009 *** (0.002)		−0.006 (0.004)		−0.010 *** (0.002)
Labor ratio		−0.010 (0.074)		−0.015 (0.059)		0.066 * (0.040)
Ln (Person income)		0.029 *** (0.008)		0.037 *** (0.007)		0.024 *** (0.009)
Ln (Person land)		0.148 *** (0.044)		0.140 *** (0.045)		0.142 *** (0.039)
Distance		−0.001 (0.004)		0.001 (0.007)		−0.003 (0.005)
Risk perception		0.015 (0.010)		0.012 (0.008)		0.015 (0.011)
Individual perception		0.003 (0.003)		−0.000 (0.005)		0.001 (0.007)
Production perception		0.003 *** (0.001)		0.010 (0.012)		0.011 (0.011)
Cost perception		−0.007 (0.007)		−0.010 (0.008)		−0.024 *** (0.009)
Severe perception		0.005 (0.005)		0.009 ** (0.005)		0.015* (0.009)
Age		−0.001 *** (0.000)		−0.001 * (0.000)		−0.000 *** (0.000)
Climate declines		−0.026 (0.034)		−0.024 (0.039)		−0.012 (0.043)
County_1 (Gaoxian)		0.017		0.007		0.015
		(0.018)		(0.010)		(0.019)
County_2 (Jiajiang)		0.072 ***		0.049 **		0.030 *
		(0.024)		(0.021)		(0.018)
Control variables	No	Yes	No	Yes	No	Yes
Regional dummies	No	Yes	No	Yes	No	Yes
Wald χ2	153.08 ***		171.17 ***		28.74 ***	
Pseudo R2	0.2292	0.3479	0.2455	0.3559	0.0935	0.1966
N	540	540	540	540	540	540

Note: N = 540; The standard errors of cluster at the county are in parentheses; The report result is marginal effect; * *p* < 0.1, ** *p* < 0.05, *** *p* < 0.01.

**Table 4:** Average treatment effects of different matching algorithms.

Matching Algorithms	Influencing Factors	ATT	Std. Err.	Treated	Controls
Nearest neighbor matching (1:4)	Relatives and friends	0.433 ** (6.33)	0.081	0.962	0.529
	Strong ties	0.300 (5.51)	0.066	0.971	0.671
	Weak ties	0.175 *** (5.11)	0.054	0.964	0.789
Radius matching (caliper 0.01)	Relatives and friends	0.446 ** (6.48)	0.073	0.961	0.515
	Strong ties	0.329 (6.14)	0.056	0.971	0.642
	Weak ties	0.173 *** (5.06)	0.039	0.962	0.789
Kernel-based matching (bandwidth 0.06)	Relatives and friends	0.376 ** (6.08)	0.067	0.962	0.583
	Strong ties	0.312 (6.24)	0.049	0.971	0.659
	Weak ties	0.183 *** (5.74)	0.035	0.964	0.781

Note: Numbers of t-values are in parentheses; ** means *p* < 0.05 and *** means *p* < 0.001.

**Table 5:** Heterogeneity analysis.

Variable	Whether the Respondents Have a Primary Education or Above?						Did the Crop Yield Decrease Due to the Weather?					
Relatives and friends	3.312 ***			3.495 ***			2.244 ***			1.751 ***		
	(0.846)			(0.944)			(0.048)			(0.339)		
Strong ties		3.448 ***			3.417 ***			4.570 ***			3.481 ***	
		(0.640)			(0.365)			(1.001)			(0.533)	
Weak ties			1.724 ***			2.234 ***			2.781 ***			1.943 ***
			(0.840)			(0.654)			(0.523)			(0.484)
N	324	324	324	126	126	126	161	161	161	379	379	379
County	Yes	Yes	Yes	Yes	Yes	Yes	Yes	Yes	Yes	Yes	Yes	Yes
Control	Yes	Yes	Yes	Yes	Yes	Yes	Yes	Yes	Yes	Yes	Yes	Yes

Note: The standard errors of the cluster at the county are in parentheses; *** *p* < 0.01; The answer on the left side of the question is “No” and the answer on the right side is “Yes”.

**Table 6:** Mechanism analysis.

Variable	Mechanism 1: Peer Effects → Social Networks → Response to Climate Change						Mechanism 2: Peer Effects → Social Trust → Response to Climate Change					
	Model 1			Model 2			Model 3			Model 4		
	X→Y	X→M	X→M→Y	X→Y	X→M	X→M→Y	X→Y	X→M	X→M→Y	X→Y	X→M	X→M→Y
Relatives and friends	3.687 ***	0.517 *	3.669 ***	3.687 ***	−0.383	3.669 ***	3.687 ***	0.826 ***	3.716 ***	3.687 ***	−0.436 **	3.824 ***
	(0.520)	(0.267)	(0.494)	(0.520)	(0.562)	(0.512)	(0.520)	(0.247)	(0.531)	(0.520)	(0.140)	(0.379)
Strong ties	3.481 ***	0.338 ***	3.470 ***	3.481 ***	−0.210	3.472 ***	3.481 ***	0.315	3.476 ***	3.481 ***	−0.597 *	3.746 ***
	(0.533)	(0.120)	(0.587)	(0.533)	(0.249)	(0.567)	(0.533)	(0.227)	(0.510)	(0.533)	(0.333)	(0.473)
Weak ties	2.116 ***	0.473 **	2.038 ***	2.116 ***	−0.606 **	2.103 ***	2.116 ***	0.261	2.095 ***	2.116 ***	0.261	2.104 ***
	(0.317)	(0.238)	(0.313)	(0.317)	(0.210)	(0.326)	(0.317)	(0.371)	(0.328)	(0.317)	(0.371)	(0.355)
Control	Yes	Yes	Yes	Yes	Yes	Yes	Yes	Yes	Yes	Yes	Yes	Yes
County	Yes	Yes	Yes	Yes	Yes	Yes	Yes	Yes	Yes	Yes	Yes	Yes

Note: The standard errors of the cluster at the county are in parentheses, and the estimation results of other control variables are slightly limited by space. * *p* < 0.1, ** *p* < 0.05, *** *p* < 0.01.

**Table 7:** Robustness test of farmers’ adaptive behavior to climate change.

	County 1 (Gao Xian)			County 2 (Jia Jiang)			County 3 (Yue Chi)		
Relatives and friends	0.167 ***			0.244 ***			0.199 ***		
	(0.039)			(0.038)			(0.046)		
Strong ties		0.194 ***			0.315 ***			0.133 ***	
		(0.040)			(0.056)			(0.035)	
Weak ties			0.092 **			0.210 ***			0.131 ***
			(0.046)			(0.057)			(0.040)
Control variables	Yes	Yes	Yes	Yes	Yes	Yes	Yes	Yes	Yes
Wald χ2	27.62 **	36.43 ***	14.98	54.92 ***	67.39 ***	33.82 ***	52.60 ***	41.72 ***	41.00 ***
Pseudo R2	0.2675	0.3528	0.1451	0.4373	0.5366	0.2693	0.5094	0.4041	0.3971
N	180	180	180	180	180	180	180	180	180

Note: The standard errors are in parentheses; ** *p* < 0.05, *** *p* < 0.01.

## Data Availability

Not applicable.
